# Post-Translational Modifications of H2A Histone Variants and Their Role in Cancer

**DOI:** 10.3390/cancers10030059

**Published:** 2018-02-27

**Authors:** David Corujo, Marcus Buschbeck

**Affiliations:** 1 Josep Carreras Leukaemia Research Institute (IJC), Campus ICO-Germans Trias i Pujol, Universitat Autònoma de Barcelona, 08916 Badalona, Spain; dcorujo@carrerasresearch.org; 2 PhD Programme of Genetics, Universitat de Barcelona, 08007 Barcelona, Spain; 3 Program for Predictive and Personalized Medicine of Cancer, Germans Trias i Pujol Research Institute (PMPPC-IGTP), 08916 Badalona, Spain

**Keywords:** histone variants, post-translational modifications, cancer, epigenetics, H2A.Z, H2A.X, macroH2A

## Abstract

Histone variants are chromatin components that replace replication-coupled histones in a fraction of nucleosomes and confer particular characteristics to chromatin. H2A variants represent the most numerous and diverse group among histone protein families. In the nucleosomal structure, H2A-H2B dimers can be removed and exchanged more easily than the stable H3-H4 core. The unstructured N-terminal histone tails of all histones, but also the C-terminal tails of H2A histones protrude out of the compact structure of the nucleosome core. These accessible tails are the preferential target sites for a large number of post-translational modifications (PTMs). While some PTMs are shared between replication-coupled H2A and H2A variants, many modifications are limited to a specific histone variant. The present review focuses on the H2A variants H2A.Z, H2A.X, and macroH2A, and summarizes their functions in chromatin and how these are linked to cancer development and progression. H2A.Z primarily acts as an oncogene and macroH2A and H2A.X as tumour suppressors. We further focus on the regulation by PTMs, which helps to understand a degree of context dependency.

## 1. Introduction

**Figure 1:**
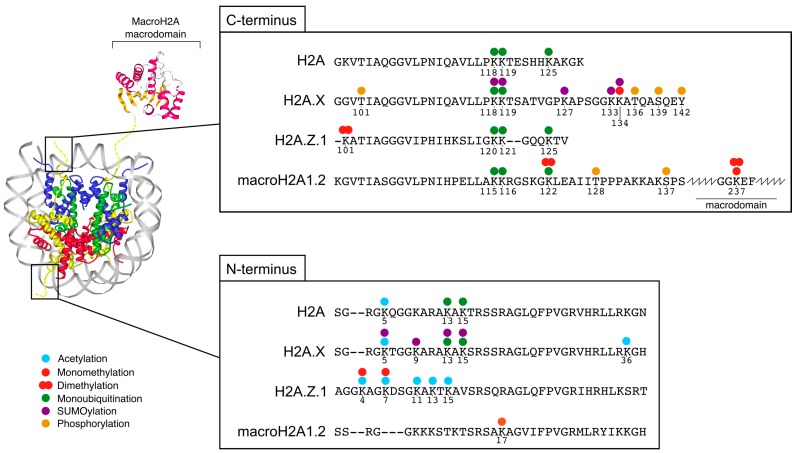
Amino acid sequence of the N- and C-termini of human H2A variants and their post-translational modifications. Alignment of human H2A type 1 (NCBI ID accession number NP_003501.1), H2A.X (NP_002906.1), H2A.Z.1 (NP_002907.1) and macroH2A1.2 (NP_004884.1). The crystal structure of a nucleosome containing a macroH2A histone is depicted. Histone H2A is colored in yellow, H2B in red, H3 in blue, H4 in green and DNA in grey. Dashed lines represent the H2A tails. The macrodomain of macroH2A is colored by secondary structure (α-helices in magenta and β -sheets in orange). The picture of the nucleosome is based on protein data bank ID 3REH [20] and generated with ProteinWorkshop [21], the picture of the macroH2A macrodomain is based on PDB ID 2FXK [22] and generated with NGL viewer [23,24].

The nucleosome particle is the basic unit of the chromatin fibre. The nucleosome consists of a histone octamer core around which 147bp of DNA are wrapped about 1.7 times. The protein core of the nucleosome is composed of the so-called core histones: H2A, H2B, H3, and H4 [1]. In particular, two units each of H3 and H4 form a tetramer around which bind two dimers of H2A-H2B (Figure 1). Additionally, the H1 linker histone binds the nucleosome and the extra-nucleosomal DNA, stabilising chromatin structure [2]. Histones are highly conserved and essential in all eukaryotes. Core and linker histones can undergo several post-translational modifications, such as methylation, acetylation, phosphorylation, ubiquitination, and SUMOylation [3,4]. In particular, covalent modifications of core histones have the potential to alter the properties of the nucleosome or the ability of protein effectors to interact with it, thus acting as essential regulators of chromatin function [5]. The extensive repertoire of histone modifications allows for the complex control and modulation of chromatin, which ensures robust gene expression patterns throughout development and cell differentiation.

The bulk of the histone pool in the cell is composed of “canonical” or replication-coupled histones, which are encoded in tandem array gene clusters and synthesized and deposited into chromatin in a replication-dependent manner. A smaller fraction of the histone pool is constituted by histone variants, which diverge to different extents in their primary sequence from their replication-coupled counterparts [6]. Histone variants are generally expressed from single-copy genes and are deposited into chromatin independently of replication by specialized machinery [7,8]. Histone variants are dynamically regulated both in their expression level and their genomic deposition. The replacement of replication-coupled core histones by histone variants provides chromatin with specific characteristics and can thus influence all functions occurring on the chromatin template including transcription and DNA repair [8,9]. This occurs through different mechanisms that include: alteration of the biophysical properties of the nucleosome [10], promoting the deposition of certain histone modifications or recruitment of specific interactors [8]. 

In humans, several variant forms have been described for H2A, H2B, and H3, some of them are “universal” and highly conserved among all eukaryotes, while others have evolved specifically in higher eukaryote lineages [11]. H2A variants represent the largest and most diverse family of histones; in human somatic cells, eight variants of H2A have been identified: H2A.X, H2A.Z.1, H2A.Z.2.1, H2A.Z.2.2, H2A.Bbd, macroH2A1.1, macroH2A1.2, and macroH2A2 [12]. Additionally, several germ cell-specific histones have been identified that function in spermatogenic differentiation and paternal genome activation after fertilisation [12,13,14,15]. This review focuses on the function of the H2A variants H2A.Z, macroH2A, and H2A.X, and their post-translational modifications in mammals, with particular emphasis on their known and potential roles in human cancer. The role of other histone variants and functions that are related to diseases other than cancer are reviewed elsewhere [2,6,8,15].

## 2. Features and Post-Translational Modifications of H2A.Z, H2A.X and macroH2A

Post-translational modifications (PTMs) are an essential and highly dynamic mechanism for the regulation of protein function and the transduction and integration of signals in the cell [16]. This is remarkably relevant for histone proteins as their covalent modifications contribute to the molecular basis of epigenetic regulation and cellular memory. Unstructured N- and C-termini of H2A variants protrude out of the core structure of the nucleosome, while for the other histones, this is only the case for their N-termini and their C-termini are structured buried in the nucleosome core (Figure 1). Given their accessible position, it is not surprising that they are the main sites for PTMs (Table 1). Some modified amino acids are shared by replication-coupled H2A and its variants, while others are specific for a certain histone type. Differences in the modification pattern are frequently driven by the differences in amino acid sequence between H2A and H2A variants, which affect the presence of PTM target residues or lead to differential recognition by chromatin modifying enzymes (Figure 1). For example, macroH2A proteins lack some of the residues that are modified in other H2As, but are modified on their unique C-terminal domain. Shared PTMs among H2A and its variants include C-terminal monoubiquitination of lysine K119 or its homologous positions [17] and the acetylation of N-terminal lysines by the TIP60 acetyltransferase complex [18,19].

In this section, we will discuss H2A.Z, macroH2A and H2A.X individually, outlining their main molecular and biological functions. We highlight current knowledge on how PTMs are regulated by enzymes (writers and erasers) and how they impact the function of the histone variant.

### 2.1. H2A.Z—A Dynamic Protein Regulated by PTMs

H2A.Z is a highly conserved variant that shares a 60% identity with H2A and is essential for early mammalian development [82]. The removal of H2A.Z results in embryonic lethality in mice, while mice that are deficient for other H2A variants complete development without major abnormalities [8,82]. Vertebrates have two H2A.Z genes that encode similar proteins, H2A.Z.1 and H2A.Z.2, which differ by three residues [83]. Alternative splicing gives rise to the additional isoform H2A.Z.2.2, which occurs mainly in the brain of primates [84]. H2A.Zs are thought to act as “dynamiser” agents that can increase chromatin accessibility and facilitate transitions between chromatin states [8]. Indeed, H2A.Z has been implicated in virtually all functions of the chromatin template, including gene transcription, DNA repair, heterochromatin formation, and chromosome segregation, with important consequences for self-renewal and differentiation of stem cells [8]. 

H2A.Z can undergo several PTMs on lysine residues that include acetylation, and methylation at the N-terminus, and methylation, ubiquitination and SUMOylation at the C-terminus [85]. The understanding of the PTMs of H2A.Z resolved the initial controversy about its role in gene regulation. Acetylated H2A.Z is preferentially found at nucleosomes near the transcription start site of active promoters, while the non-acetylated form of the protein is enriched at the entire promoter of inactive genes and heterochromatic positions [86].

H2A.Z can be extensively acetylated at five lysine residues in its N-terminal tail: K4, K7, K11, K13 and K15 [25,26,27]. In mouse embryonic stem cells (mESCs), K7 and K11 are the most frequently modified residues [27] and thought to be mainly produced by the action of H4/H2A histone acetyltransferase TIP60 [87,88]. Generally, acetylation destabilizes the nucleosome and favours an open conformation that is more permissive to the transcriptional machinery [26,89]. H2A.Z and its acetylation favour the recruitment of regulatory complexes and are an important feature of enhancer activation [27,90,91,92]. As a consequence of its involvement in gene and enhancer activity, H2A.Z acetylation is important for cellular differentiation [27]. This is well illustrated in myogenesis where a non-acetylable form of H2A.Z blocks differentiation by suppressing the expression of the transcriptional master regulator, MyoD [93].

A substantial portion of H2A.Z is monoubiquitinated on K120, K121, or K125, with K120 being the main acceptor site modified by the Polycomb repressive complex 1 containing the E3 ligase RING1B [27,31]. Similarly to H2A, monoubiquitinated H2A.Z is enriched at regions of H3K27me3-marked facultative heterochromatin and is associated with repressed transcription [31]. Several promoters in pluripotent cells are characterised by “bivalent chromatin” that contains both active H3K4me3 and repressive H3K27me3 marks, granting a state that is silent but poised for rapid activation [94]. In mESCs, H2A.Z histones modified both by acetylation and ubiquitination can be found at bivalent chromatin domains mimicking the co-occurrence of active and repressive marks known for H3 [27]. The enzyme USP10 deubiquitinates H2A.Z in transcriptional activation processes [32].

H2A.Z can be mono- or dimethylated on both N- and C-terminal lysines. N-terminal monomethylation of H2A.Z at preferentially K7 but also K4 is carried out by SETD6 and is necessary for self-renewal of mESC [33]. In the same cells, H2A.ZK7me is present at the promoter of Polycomb-repressed differentiation genes marked by H3K27me3 and both PTMs are removed upon retinoic acid-induced differentiation [33]. Additionally, lysine K101 at the C-terminus of H2A.Z can be dimethylated by the histone methyl-transferase SMYD3, hindering its eviction by the ANP32E chaperone and thus allowing a more stable association with chromatin [34,95]. Removal of H2A.Z by ANP32E is, for instance, important for chromatin remodelling at sites of DNA double-strand breaks (DSBs) [96,97], suggesting that the deposition of H2A.Z.1K101me may play a role in the dynamics of DNA repair. 

SUMOylation of H2A.Z has also been linked to DNA damage signalling. In yeast cells, a small fraction of H2A.Z is SUMOylated in its C-terminus mainly at positions K126 and K133, and this modification is required for the localization of unrepaired DSBs to the nuclear periphery [35]. After DSB induction in human cells, the E3-ligase PIAS4 SUMOylates H2A.Z.2, which increases its mobility, thus favouring its exchange [36].

In conclusion, H2A.Z is a multi-faceted histone variant whose impact on chromatin function is greatly affected by PTMs. Acetylation and SUMOylation increase the dynamic properties of H2A.Z, thereby favouring processes, such as DNA repair and transcriptional activation. In contrast, methylation and ubiquitination confer a more stable chromatin association paired with transcriptionally silenced states.

### 2.2. MacroH2A—Stabilizing Histones with a Unique Tripartite Structure

MacroH2A is the most divergent histone variant in terms of sequence and structure when compared to its replication-coupled counterpart. This atypical histone is characterised by a tripartite structure; in addition to an N-terminal histone fold, it contains an unstructured linker region and a large globular macrodomain at the C-terminus [22,98]. The histone fold of macroH2A shares around 65% identity with replication-coupled H2A [99] and does not introduce major changes to the nucleosome core particle [98]. However, a pronounced change is the drastic extension of the accessible C-terminus that contains two entire additional domains: the linker and the macrodomain [98,100].

Mice lacking all macroH2A are viable but have a retarded growth and metabolic alterations [101], while macroH2A-defficient zebrafish embryos display several developmental defects [102]. Multiple studies have implicated macroH2A proteins in differentiation, somatic cell reprogramming, and cancer [8]. The current body of evidence on macroH2A supports its function as an epigenetic stabilizer that helps to establish and maintain differentiated states. On the molecular level, this is correlated with a major role of macroH2A in maintaining nuclear organization and heterochromatin architecture [103,104]. As a global stabilizer, macroH2A might have an opposed function to H2A.Z.

The presence of a C-terminal macrodomain is the major peculiarity of macroH2A. Macrodomains are highly conserved globular folds that can bind NAD+-derived metabolites such as ADP-ribose and are also present in non-chromatin components [105]. There are three different macroH2A proteins in vertebrates: macroH2A1.1 and macroH2A1.2 arise by alternative splicing of a single gene while macroH2A2 is encoded by a separate gene [106,107,108]. The three macroH2A proteins differ in key residues in their macrodomain and only macroH2A1.1 is able to bind ADP-ribose and ADP-ribosylated proteins [22]. This particularity allows for macroH2A1.1 to bind, and, in certain contexts, inhibit ADP-ribosylated PARP1, thereby functionally impacting on PARP1-dependent processes, such as DNA-damage repair [109], stress response [110], and transcriptional regulation [111]. MacroH2A1.1 is poorly expressed in fast proliferating cells but upregulated during differentiation [112,113]. In differentiated muscle cells, macroH2A1.1 is the predominantly expressed macroH2A protein and globally impacts on cellular metabolism, including mitochondrial activity, through inhibiting PARP-1-dependent NAD+ consumption [114]. 

While the histone fold domain of macroH2A has been found monomethylated and ubiquitinated, phosphorylation and additional methylation sites have been reported on the linker and macrodomain, albeit not for all isoforms. However, knowledge on the function of these modifications remains scarce. For instance, monomethylation of macroH2A1 on K17 and dimethylation of K122 have been reported [37], but their function remains elusive. Additional methylation sites and states were found when the protein was overexpressed (Table 1) [37]. Ubiquitination of macroH2A1 occurs at sites K115/116, which correspond to conserved sites also modified in H2A [37,38]. While RING1B is considered to be the major ubiquitin E3 ligase for H2A and its variants, CULLIN3/SPOP is also able to mediate ubiquitination of macroH2A1 on the K115/116 acceptor site [39,115]. Again, similar to what has been observed for H2A and H2A.Z, ubiquitination of macroH2A is associated with its deposition in heterochromatin [39]. In addition, BRCA1 was shown to ubiquitinate macroH2A1.1 on K123, which contributes to the induction of senescence [40]. 

The linker shared by both macroH2A1 splice isoforms can be phosphorylated on T128 and S137 [37,41]. T129, but not S137, is also conserved in macroH2A2, but its potential phosphorylation has not been analysed so far [37,41]. S137-phosphorylated macroH2A1 is excluded from the inactive X chromosome and, while only a small fraction of macroH2A1 is phosphorylated, its abundance greatly increases during mitosis [41]. Both Haspin as well as cell cycle regulators Cdk1/cyclinB and Cdk2/cyclinE have been suggested to catalyse S137 phosphorylation [41,42].

While several PTMs of macroH2A1 have been identified, their function remains largely elusive and many of the involved writers and erasers still need to be identified. MacroH2A2 is poorly studied in the context of PTMs. 

### 2.3. H2A.X—The Histone Variant at the Core of the DNA Damage Response

Histone variant H2A.X shares a high amino acid similarity with replication-coupled H2A but is characterised by an extended C-terminus [116]. The levels of H2A.X range between 2% and 25% of total H2A depending on the cell type [117]. Although mice without H2A.X are viable, they display hypersensitivity to radiation [118] and have an increased genomic instability [119]. H2A.X functions mainly in the DNA damage response (DDR), a process that is strongly regulated by PTMs [120,121]. Phosphorylation and ubiquitination of H2A.X are particularly well studied as key events in the detection and response to DNA damage in the form of DSBs. Beyond the DDR, several studies have identified roles for H2A.X in X chromosome inactivation, stem cell biology and cellular senescence [122]. 

The C-terminus of H2A.X can undergo phosphorylation in three of its unique residues: T136, S139, and Y142. Phosphorylation of S139 occurs rapidly and extensively as a very early step in DDR to DSBs [117]. The S139 phosphorylated H2A.X, frequently referred to as γ-H2A.X, is an essential signal for the recruitment and retention of DDR complexes [123]. Three different PI3K-like kinases mediate this phosphorylation: ATM and DNA-PK share functional redundancy in response to ionizing radiation [45], while ATR phosphorylates H2A.X during replication stress [46]. A number of phosphatases, including PP2A, PP4, PP6, and Wip1 are able to dephosphorylate γ-H2A.X [47,48,49,50,51,52]. In addition to S139, H2A.X is also phosphorylated on T136 by DNA-PK [44] and on Y142 by WSTF/BAZ1B [54,55]. These modifications are likely to fine-tune the duration and intensity of DDR signalling by H2A.X. Indeed, the EYA phosphatase removes Y142 phosphorylation upon DSB to facilitate repair, whereas the persistency of this PTM triggers apoptosis [56]. Additionally, residue T101 is phosphorylated upon ionizing radiation exposure and is required for the H2A.X-mediated survival [43]. 

Besides phosphorylation, ubiquitination of H2A.X (and of replication-coupled H2A) is critical for DNA repair by acting as a signal and binding platform for DDR factors [124,125]. Monoubiquitination of H2A.X occurs at K119 and K120 in its C-terminus, homologous to H2A ubiquitination at K119, by the E3 ubiquitin ligase RNF2 (RING1b/RING2) and its adaptor protein BMI1, both members of the PRC1 complex. This monoubiquitination event is an early step following DNA damage and is required for the recruitment of the kinase ATM to the damaged site, which allows rapid phosphorylation of H2A.X on S139 [71,72,73,74,75]. The S139 phosphorylation is recognized by the adaptor protein MDC1 which further recruits the E3 ubiquitin ligases RNF8 [126,127,128,129,130] and RNF168 [57,58]. RNF168 directly monoubiquitinates H2A.X and H2A at residues K13 and K15, which together with H4K20me3 permits the recruitment of 53BP1, a factor that favors repair by non-homologous end joining in G1 over homologous recombination (HR) through the inhibition of BRCA1 [59,60,61,62,70]. RNF8 further potentiates RNF168 recruitment through ubiquitination of the H1 linker histone [131]. Interestingly, RNF168 is also able to ubiquitinate H2A.Z, although the modified sites and relevance in the DDR remain to be determined [30]. Several deubiquitinating enzymes are responsible for the removal of H2A.X bound ubiquitin, including USP3, USP11, USP16, USP44, Dub3, BRCA1-A complex member BRCC36 and proteasome associated deubiquitinating enzyme POH [63,64,65,66,67,68,69]. 

Less studied modifications of H2A.X are acetylation, methylation and SUMOylation, some of which also contribute to DDR by recruiting repair factors. Acetylation of K5 by TIP60 does not only facilitate ubiquitination at K119 and mobility [76,77], it is also required for the accumulation of the repair factor NBS1 and Poly-ADP-ribosyl-polymerase 1 (PARP1/ARTD1) activity [132,133]. Methylation of K134 is involved in the repair process and contributes to the recruitment of the kinases ATM and ATR [79], but the function of Suv39H2 as K134 methylase is highly controversial [80]. Another acetylation catalysed by CBP/p300 on K36 is independent of DNA damage, but might contribute to survival signalling [78]. In human cells, H2A.X can also be SUMOylated on multiple lysines, which requires PIAS4, but is not dependent on DNA damage [81].

H2A.X is a chromatin component that has a central position in the DNA damage response. Its function is mediated by several PTMs, most notably phosphorylation and ubiquitination, which are tightly interconnected and synergize in signal transduction, chromatin remodelling, and recruitment of repair factors. 

## 3. Role of H2A Variants in Cancer

Genetic and epigenetic alterations are drivers of cancer initiation and progression. In this section, we will discuss the role of histone variants H2A.Z, macroH2A, and H2A.X in cancer. Whenever known, we will highlight the involvement of their PTMs. Current understanding positions H2A.Z as having mainly oncogenic functions, while macroH2A and H2A.X are considered as tumour suppressors (Table 2). In addition, H2A.X is used as biomarker for drug response. 

### 3.1. H2A.Z Frequently Promotes Pro-Oncogenic Transcription

H2A.Z facilitates the transcriptional activation of several genes that promote cancer initiation, growth, and/or metastatic potential. Concordantly, H2A.Z overexpression is frequent and correlates with poor survival in melanoma, hepatocellular carcinoma, and breast cancer [134,142,143]. 

In particular, acetylated H2A.Z on gene promoters and enhancers drive gene expression. How this is linked with an oncogenic transcription activation is best characterised in hormone-dependent cancers [171]. In breast cancer, H2A.Z is a direct target of estrogen signalling and mediates estrogen-receptor α (ERα)-induced gene transcription on promoters and enhancers [92,134]. Moreover, the overexpression of H2A.Z promotes proliferation [135]. The requirement for the acetylation of H2A.Z has been particularly established in prostate cancer where H2A.Z plays a similar role in androgen-receptor mediated gene activation [137,138]. In addition, the deubiquitination of H2A.Z by USP10 is required for the androgen-receptor mediated transcriptional activation of hormone-regulated genes [32]. Moreover, the H2A.Z depositing chromatin remodelers SRCAP and p400 promote progression of hormone-dependent cancers [139,172,173].

Furthermore, H2A.Z also promotes progression of non-hormone-dependent cancers. In colorectal cancer, H2A.Z and p400 act downstream of Wnt signalling [174]. In osteosarcoma cells, H2A.Z mediated the induction of cell cycle genes and proliferation [175], reflecting a conserved function in yeast [176]. However, not all H2A.Z induced transcriptional events are necessarily oncogenic. For instance, histone deacetylase inhibitors induced the expression of the cell cycle inhibitor p21 in ER-negative p53^−/−^ breast cancer cells, presumably by boosting H2A.Z acetylation [177]. Controversial observations have linked H2A.Z to epithelial-mesenchymal transition (EMT), a process that is essential for early development and metastasis [178]. In hepatocellular carcinoma cells, H2A.Z.1 promoted EMT [142], whereas in canine kidney cells, H2A.Z kept EMT at bay by facilitating epithelial gene expression and inhibiting mesenchymal genes [179].

The methylation of H2A.Z is potentially important in cancer. The histone methyltransferase SMYD3 is upregulated in several types of cancer and promotes proliferation [136,180,181,182,183]. Only recently SMYD3 has been linked to H2A.Z: In breast cancer cells, dimethylation of H2A.Z at K101 by SMYD3 promotes cell proliferation at least in part by activating cyclinA1 expression [34]. SETD6, the enzyme that is responsible for H2A.Z methylation at K4 and K7, acts as a co-activator for estrogen-induced genes and its silencing reduces proliferation in breast cancer cells [184]. However, a possible dependence of this effect on the methylation status and dynamics of H2A.Z has not been investigated. 

In conclusion, H2A.Z is a histone variant that frequently favours oncogenic events, especially through facilitating the activation of genes and regulatory elements. In this regard, acetylation and deubiquitination of H2A.Z play an essential role in permitting transcriptional activation of oncogenes. Methylation and SUMOylation of H2A.Z could potentially have the opposite effect in this process, but their implication and relevance in the context of cancer still need to be explored.

### 3.2. MacroH2A Is a Context-Dependent Tumour Suppressor

MacroH2A histones are generally regarded as having a tumour suppressive function in cancer. MacroH2A expression decreases as the disease progresses in melanoma, bladder cancer, and anal neoplasms [145,148,155]. Knocking-down macroH2A results in more aggressive teratomas, melanoma, breast cancer, and bladder cancer [113,145,149,152], while its overexpression reduces proliferation in several cancer cell lines [145,152,153].

However, there are important differences in the effect of the different isoforms of macroH2A in cancer [185]. In particular, the expression level of macroH2A1.1 is inversely correlated with proliferation and its low levels are a marker for poor prognosis in lung and colon cancer [112,153,154,186]. In contrast, macroH2A1.2 expression is generally higher in highly proliferative cancer cell lines [111] and a high macroH2A1.2 to macroH2A1.1 ratio increases migration, invasion, and growth of breast cancer cell lines [152,187]. In triple negative breast cancer, however, the level of macroH2A1.1 expression correlates with poor survival and EMT [188]. Other evidences support a tumour suppressive role of macroH2A1.2 in melanoma and bladder cancer [145,149], suggesting that macroH2A1.2 function might be strongly context-dependent. The splicing factors MBNL1 and QKI favour macroH2A1.1 expression over macroH2A1.2 [112,153], while the RNA helicases Ddx5 and Ddx17 do the opposite [152]. Expression of MBNL1 and QKI is altered in various cancer types [189]. In particular, the expression of QKI limits proliferation in gastric cancer at least in part through promoting macroH2A1.1 expression [186].

MacroH2A has an ambiguous role in transcription; while contributing to repression, it also participates in signal-induced gene activation [190,191]. Thus, macroH2A is involved in the transcriptional control of oncogenes, tumour suppressors, and cell cycle regulators. To give a few examples: MacroH2A represses the expression of CDK8 in melanoma cells, which limits their proliferative potential [145,146]. MacroH2A1 represses rDNA gene clusters that are upregulated in highly proliferative cells [192]. In a colorectal cancer cell line, however, macroH2A1 together with DNA methylation lead to the repression of the cell cycle inhibitor p16, which results in higher proliferation [193]. Possibly through its capacity to bind PARP-1, macroH2A1.1 positively regulates the level of tumour suppressive SOD3 and the gene expression program that supports paracrine senescence [152,194]. 

Loss of macroH2A1 leads to the acquisition of stem-like features and gene expression programs, which include LIN28A transcription in bladder cancer and increased NF-Kb signalling in hepatocellular carcinoma [149,195]. This observation is reminiscent of the function of macroH2A in limiting epigenetic plasticity during stem cell differentiation and as barrier to somatic cell reprogramming [8]. 

Genomic regions that are particularly sensitive to replication stress are termed fragile sites and are a frequent source of genomic instability in cancer cells [196,197]. A recent study shows that macroH2A1.2 accumulates at fragile genomic regions and promotes BRCA1 recruitment at stalled replication forks, protecting against replication stress and damage-induced senescence [198]. Furthermore, macroH2A is recruited to DNA damage sites where it promotes chromatin compaction and BRCA1 recruitment [199,200].

In conclusion, macroH2A is a differentiation-promoting factor that limits the acquisition of malignant characteristics by cancer cells. While current evidence clearly supports a tumour suppressive role for macroH2A1.1 and macroH2A2, the function of macroH2A1.2 seems to depend greatly on the context of the particular cancer studied. Remarkably, the regulation of macroH2A by PTMs remains virtually unknown and experimental work in this direction will likely help to understand the context-dependent effects of this histone variant in cancer.

### 3.3. H2A.X Is a Tumour Suppressor and a Biomarker

If not properly repaired, DNA damage can promote genomic instability, which is associated with pre-malignant stages and drives cancer progression [201]. At the same time, the induction of high levels of DNA damage is an effective treatment strategy to kill cancer cells [202]. H2A.X and, in particular, the S139-phosphorylated form γ-H2AX, has a pivotal role in the DNA damage response. Thus, H2A.X has a dual role in cancer as a tumour suppressor factor preventing genome instability and as a biomarker monitoring treatment-induced DNA damage [203,204].

Mice without H2A.X are hypersensitive to radiation [118] and have impaired DNA repair and increased genomic instability that leads to increased susceptibility to develop T and B cell lymphomas in a p53 deficient background in a dosage dependent manner [119,156]. Mutations and deletions in the 11q chromosomal arm region that contains the H2A.X gene, as well as other important DNA damage response factors, like ATM, happen frequently in cancers such as sporadic breast cancer [157], neuroblastoma [158], head and neck squamous cell carcinomas [160] and in hematopoietic malignancies like chronic lymphocytic leukaemia [163,164]. A recent study identified ZMYM3 as a novel DDR factor frequently mutated in cancer that specifically binds to H2A.X and macroH2A histones and maintains genome integrity through BRCA1 function modulation [205]. These and other studies remark that H2A.X and proper DSB repair are essential tumour suppressive mechanisms.

The immunodetection of S139 phosphorylated H2A.X is used to quantify DNA damage in cells and tissues, and has diagnostic and prognostic value in cancer. High phosphorylation levels are indicative of defective DNA repair and genomic instability in premalignant lesions and tumours and are associated with higher malignancy and poor prognosis in various cancers, including breast, colorectal, lung, ovarian, and melanoma [206]. In addition, S139-phopshorylated H2A.X is also proposed to serve as a predictive biomarker for the risk of hepatocellular carcinoma in preneoplastic lesions [207]. Interestingly, S139-phospho levels in peripheral blood have risk prediction value in bladder and colorectal cancer and could serve as a non-invasive biomarker [208,209,210]. As many chemotherapy and radiotherapy treatments for cancer aim to induce DNA damage in cancer cells, detection of S139-phosphorylated H2A.X is used in many clinical studies to measure the effects of treatment in patients and to evaluate individual radiosensitivity [211,212]. 

Ubiquitination of H2A.X is less studied in cancer but the E3 ligases RNF168 and RNF8 have been implicated in cancer and genomic instability. Mice deficient in RNF8 or RNF168 suffer from increased radiosensitivity, genomic instability, and tumorigenic risk [213,214]. In breast cancer, chemotherapy-induced oxidative stress enhances H2A.X ubiquitination by RNF168 which reduces H2A.X levels in tumour cells, making them more sensitive to genotoxic stress and improving patient survival [168]. In contrast, RNF8 is a co-activator of the estrogen receptor and promotes cell growth and EMT in breast cancer cells, although this mechanism has not yet been linked to H2A.X ubiquitination [215,216].

As a conclusion, H2A.X is an essential component of the cellular machinery that controls and repairs DNA damage. Its proper regulation is required to safeguard genomic stability and to prevent neoplastic growth and malignant disease progression. S139 phosphorylation of H2A.X is at the same time a functionally critical component of the repair process and a promising marker in cancer for risk assessment, early detection, prognosis, and treatment evaluation.

## 4. Outlook and Conclusions

The substitution of replication-coupled H2A by one of its variants represents an epigenetic regulation mechanism that impacts virtually all of the processes occurring in chromatin, and is thus highly relevant in many physiological contexts. Deregulation of the function of histone variants is therefore crucial in many aspects of tumour origin and progression. Beyond the functional specificity conferred by each histone variant sequence variations, several PTMs expand the functional diversity contained in this protein family. While much progress has been made in understanding the major role of some of these PTMs, many of them are still poorly described and their functional relevance remains unknown. Moreover, little is known about to which extent the co-occurrence of PTMs in the same histone variant happens and which combinatorial effects and functional cross-talks could be derived from this fact [217]. In particular, it will be essential to identify reader proteins that discriminate between histone variants and their different PTMs. Advances in the proteomics field and in particular the sensitivity of quantitative mass spectrometric analysis will surely be an essential factor to progress in this regard [218]. 

The deposition and removal of many PTMs is tightly linked to cellular metabolism through the availability of key metabolites, effectively coupling the metabolic status of the cell with epigenetic regulation mechanisms that involve PTMs [219,220,221]. Recent work has shed light into this interplay particularly for DNA and histone methylation and histone acylations, which include acetylation and other modifications that are not covered in the present review [222,223]. Cancer cells display many metabolic alterations that are the subject of various therapeutic strategies [224]. This altered metabolic state potentially connects with reshaped PTM profiles that could impact transcriptional events, promoting disease progression [225]. Further study into this crosstalk will possibly reveal new therapeutic opportunities.

A better understanding of the function of H2A variants will contribute to our knowledge on epigenetic regulation, and, most importantly, provide insight into alterations that happen in diseases such as cancer.

## Figures and Tables

**Table 1:** H2A variants and their Post-translational modifications (PTMs).

Histone	Modification	Residues	Writers	Erasers	Readers	References
H2A.Z	Acetylation	K4, K7, K11, K13, K15	TIP60	Uncharacterised histone deacetylases (HDACs)	BPTF, Brd2	[19,25,26,27,28,29]
	Monoubiquitination	Unknown	RNF168	Unknown	Unknown	[30]
	Monoubiquitination	K120, K121, K125	RING1B (PRC1)	USP10	Unknown	[27,31,32]
	Methylation	K4, K7	SETD6, SMYD3	Unknown	Unknown	[33]
	Dimethylation	K101	SMYD3	Unknown	Unknown	[34]
	SUMOylation	K126, K133 (Yeast)	PIAS4	Unknown	Unknown	[35,36]
MacroH2A	Monoubiquitination	K115, K116	PRC1, CULLIN3/SPOP	Unknown	Unknown	[17,37,38,39]
	Monoubiquitination	K122	BRCA1	Unknown	Unknown	[40]
	Methylation	K17 (mono), K122 (di), K237 (mono/di)	Unknown	Unknown	Unknown	[37]
	Phosphorylation	T128	Unknown	Unknown	Unknown	[37]
	Phosphorylation	S137	Cdk1/cyclinB, Cdk2/cyclinE, Haspin kinase	Unknown	Unknown	[41,42]
H2A.X	Phosphorylation	T101	Unknown	Unknown	Unknown	[43]
	Phosphorylation	T136	DNA-PK	Unknown	Unknown	[44]
	Phosphorylation	S139	ATM, ATR, DNA-PK	PP2A, PP4, PP6, Wip1	MDC1	[45,46,47,48,49,50,51,52,53]
	Phosphorylation	Y142	WSTF	EYA	Impairs MDC1	[53,54,55,56]
	Monoubiquitination	K13, K15	RNF168	USP3, USP11, USP16, USP44, BRCC36, Dub3	53BP1	[57,58,59,60,61,62,63,64,65,66,67,68,69,70]
	Monoubiquitination	K118, K119	RNF2-BMI1 (PRC1)	Unknown	Unknown	[71,72,73,74,75]
	Acetylation	K5	TIP60	Uncharacterised HDACs	Unknown	[76,77]
	Acetylation	K36	CBP/p300	Unknown	Unknown	[78]
	Methylation	K134	SUV39H2	Unknown	Unknown	[79,80]
	SUMOylation	K5, K9, K13, K15, K118, K119, K127, K133, K134	PIAS4	Unknown	Unknown	[81]

**Table 2:** The function of H2A variants in cancer.

Histone	Cancer Type	Alteration	Observation/Function	PTM Involvement	References
H2A.Z	Breast	Upregulation	Correlates with poor survival, promotes proliferation, involved in gene activation by estrogen signalling	Dimethylation on K101 promotes proliferation by activating cyclinA1 H2A.Z acetylation may be responsible for increased p21 expression in ER-negative p53^−/−^ breast cancer cells	[34,92,134,135,136]
	Prostate	Upregulation	Involved in gene activation by androgen signalling, poises PSA activation	Acetylation and deubiquitination are necessary for oncogenic hormone-mediated activation	[32,137,138,139,140]
	Bladder	Upregulation	Promotes cell proliferation and oncogene transcription by recruiting WDR5 (MLL complex) and BPTF (NuRD complex)	Unknown	[141]
H2A.Z.1	Hepatocellular carcinoma	Upregulation	Correlates with poor survival, promotes tumour growth and EMT	Unknown	[142]
H2A.Z.2	Melanoma	Upregulation	Correlates with poor survival, its depletion sensitizes cells to chemotherapy	Unknown	[143,144]
macroH2A1, macroH2A2	Melanoma	Downregulation	Promotes disease progression and metastasis	Unknown	[145,146,147]
macroH2A1, macroH2A2	Bladder cancer	Downregulation	Correlates with disease progression, promotes cell growth, stemness and invasiveness	Unknown	[148,149]
macroH2A1	Hepatocellular carcinoma	Upregulation	Higher immunopositivity in steatosis-associated hepatocellular carcinoma, prevents chemotherapy-induced senescence	Unknown	[150,151]
macroH2A1.1, macroH2A1.2	Breast cancer	Increased macroH2A1.2/macroH2A1.1 ratio	Observed in highly proliferative tumours, correlates with poor survival, promotes tumour growth and metastasis	Unknown	[152]
macroH2A1.1	Colorectal cancer	Downregulation	Correlates with poor survival, promotes proliferation and metastasis	Unknown	[112]
macroH2A1.1	Lung cancer	Downregulation	Correlates with higher risk of tumour recurrence	Unknown	[153,154]
macroH2A2	Anal neoplasm	Downregulation	Correlates with disease progression	Unknown	[155]
H2A.X	Sporadic breast cancer	Deletion	Proposed to increase genomic instability and tumorigenesis as observed in KO mice	Does not apply	[118,119,156,157]
	Neuroblastoma	Deletion	Correlates with disease progression and poor prognosis	Does not apply	[158,159]
	Head and neck squamous cell carcinoma	Deletion	Associated with higher genomic instability and reduced radiosensitivity, included in predictive model for recurrence and metastasis risk	Does not apply	[160,161,162]
	Chronic lymphocytic leukaemia	Deletion	Associated with higher genomic instability, correlates with poor prognosis	Does not apply	[163,164]
	Triple negative breast cancer	Upregulation	High levels of γ-H2A.X correlate with poor prognosis	S139 Phosphorylation	[165]
	Melanoma	Upregulation	High levels of γ-H2A.X observed in melanocytic lesions	S139 Phosphorylation	[166,167]
	Breast cancer	Downregulation	Correlates with better prognosis	Chemotherapy induces H2A.X degradation mediated by polyubiquitination at K13 and K15	[168]
	Colon cancer cells	Downregulation	Promotes EMT	Unknown	[169]
	Breast cancer cells	Downregulation	Promotes EMT	Unknown	[170]
